# The Reliability and Validity of Dental Indifference Scale among Romanian Young Adults

**DOI:** 10.3390/healthcare11060876

**Published:** 2023-03-17

**Authors:** Ruxandra Sfeatcu, Beatrice Adriana Balgiu, Andreea Băluță, Bogdan Mihai Gălbinașu, Carmen Elena Georgescu, Roxana Romanița Ilici

**Affiliations:** 1 Department of Oral Health and Community Dentistry, Faculty of Dentistry, Carol Davila University of Medicine and Pharmacy, 17-21 Calea Plevnei Street, 010221 Bucharest, Romania; 2 Department of Career and Educational Training, University Politehnica of Bucharest, 313 Splaiul Independenţei, 060042 Bucharest, Romania; 3 Department of Esthetics in Dental Medicine, Faculty of Dentistry, Carol Davila University of Medicine and Pharmacy, 17-21 Calea Plevnei Street, 010221 Bucharest, Romania; 4 Department of Teeth and Dental Arches Morphology and Dental Materials, Faculty of Dentistry, Carol Davila University of Medicine and Pharmacy, 17-21 Calea Plevnei Street, 010221 Bucharest, Romania

**Keywords:** dental indifference, reliability, validity, oral health

## Abstract

The study aimed at examining the reliability and the validity of the Dental Indifference Scale (DIS), which measures the significant undervaluing attitude towards the state of one’s oral health. The study has a cross-sectional design in which 660 young Romanian adults (Mean_age_ = 30.69; 30.30% males) completed an online survey in which the Dental Indifference Scale was included alongside five items related to one’s behavior towards oral health. The reliability was calculated by means of internal consistency and test-retest after two or three weeks. The DIS scores were associated with the questions regarding oral health habits. Although DIS is discriminatory regarding the behavior towards oral hygiene (brushing, flossing) and diet, the reliability of the scale is low (α = 0.37; ω = 0.39; Intraclass correlation coefficient_test-retest_ = 0.60). In comparison with prior research, no gender differences were found. In exchange, the scores for dental indifference (DI) are significantly different when it comes to comparing people with secondary education and people who are university graduates. The study shows that DIS needs to be used with caution and only with other instruments that evaluate attitudes and behaviors related to oral health which passed the test of validation in various cultural models, the Romanian one included.

## 1. Introduction

Dental indifference was conceptualized as a significant undervaluing attitude towards one’s dental health and disinterest in maintaining one’s oral health [1]. Dental indifference entails the neglect of oral health, the non-observance of recommendations regarding oral health, and the quick fix of oral health issues (for example, the individual prefers having a tooth taken out to treating it) [1]. Dental indifference was shown to be a reason for missing dental appointments, with the suggestion that the levels of indifference are related to the social and the personal circumstances in which people live [1].

Evidence shows that, in general, dental neglect can result in various oral health issues, such as periodontitis [2,3,4], as well as general diseases such as rheumatoid arthritis, pharyngitis and osteomyelitis [5]. The assessment of dental indifference as a refusal of dental treatment was associated with treatment non-adherence [6], which has negative consequences on the individual’s health and on one’s quality of life [7].

The following rationales were used to determine the psychometric analysis of an instrument that measures dental indifference: because dental negligence means poor health and reduced quality of life, it follows that there is a need to adapt robust measures in order to carry out evaluations and research in this sense. Lastly, the fact that young people are characterized by an attitude and behavior of greater dental indifference than mature people [8] makes us analyze DIS [1] specifically with regard to this population category. From this perspective, the study included the translation of DIS into Romanian, therefore the cultural and the linguistic adaptation of the DIS instrument and the evaluation of the psychometric properties of the Romanian version.

## 2. The Psychometric Properties of the Dental Indifference Scale

The Dental Indifference Scale contains eight questions; and each question has between three and five choices in the case of seven questions and eight choices in the case of one question. There are 33 items in total. The multiple choices for every statement generate a score of a maximum 1 point per question and between 0 and 8 points as a total score. The higher the score, the higher the level of dental indifference. According to Nuttall (1996), the levels of dental indifference can be divided into low (0–1), average (2–4), and high (5–8) [1]. Young people are more likely to have higher scores of dental indifference [1,8]. In addition, dental indifference was associated with tooth loss. On average, those who obtained a high score for DIS had fewer teeth than the rest of the individuals with low DIS scores [1].

The levels of dental indifference were identified from the point of view of the education level as well: individuals with primary education have high scores (Mean dental indifference score = 3.4; Std.Dev. = 1.9), unlike those with tertiary education (Mean dental indifference score =2.7; Std.Dev. = 1.7). The low education level was found to be a risk factor for the dental indifference towards treatment, and oral health in the case of people in the 45 to 65 age bracket indicated higher scores of dental indifference (Mean = 4.0; Std.Dev. = 1.8) in young people (18–34 years old), in comparison with more mature people (Mean = 3.1; Std.Dev 1.7; for the 35 to 44 age bracket; Mean = 2.6; Std.Dev. = 1.7; for the 45 to 64 age bracket) and with the elderly (Mean = 2.5; Std.Dev. = 1.2—over 65) [8,9]. At the same time, dental indifference is higher for individuals who go to the dentist only when they face dental issues, unlike those who go to regular dental check-ups and those who utilize dental services very rarely, such as every five years [8].

The scale put forth contradictory data on reliability. While Nuttall (1996) found 0.71 for α and 0.79 for test-retest reliability in the case of 910 Scottish adults [1], Skaret et al. (2000) found a Pearson correlation coefficient of 0.43 for test-retest after 15 weeks for the eight questions and an α coefficient of 0.35 for internal consistency in the case of a Norwegian population [10]. Other studies found an α Cronbach coefficient for DIS of 0.91 in the case of a population in Peru in the 18 to 45 age bracket [11].

Skaret et al. (2000) analyzed the validity of DIS by associating the scores for DIS (DIS-sum-scores) with how frequently individuals miss dental appointments [10]. The intensity of the correlation in the case of patients who missed the appointments is r = 0.46, unlike the correlation obtained in the case of the whole group (r = 0.24) [10].

Studies have also shown that, due to scores of a maximum 1 point per question, neither concurrent validity nor factorial analysis can be carried out in the case of DIS [8]. DIS was compared with another scale that measures the failure to maintain oral health and the physical neglect of one’s mouth, the Dental Neglect Scale (DNS). Although the two instruments have similar associations with socio-demographic variables, there is still a moderate degree of agreement between the two instruments, and the association between the scores of the two scales is moderate as well (r = 0.58; *p* < 0.01). Therefore, the concept of dental indifference differs from the one of dental neglect [8].

## 3. Materials and Methods

### 3.1. The Translation and the Adaptation of the Scale

The scale was translated in accordance with the recommendations of the World Health Organization (2020) by using the forward-backward translation method [12]. In the first stage, the original version of DIS in the English language was translated into Romanian by the authors of the present study. We made sure that the Romanian equivalents of each and every item would not modify the meaning of the answer scale. In the next stage, after refining the Romanian formulations, an independent translator translated the instrument from Romanian into English. The comparison of the two versions led to insignificant modifications of the items. Finally, we reached the version that we used for the study (Table A1).

### 3.2. Ethical Consideration

The study was approved by the Ethical Commission of “Carol Davila” University of Medicine and Pharmacy, Bucharest (Protocol No. 28447/18.10.2021). The study has been conducted in full accordance with ethical principles, including the World Medical Association Declaration of Helsinki of 1975 (and revised in 2013).

### 3.3. Study Design and Participants

The research had a cross-sectional design. The data were collected between October and November 2021 by sharing the research instrument set in a Google Forms specific link on social networks (Facebook and WhatsApp). The snowball sampling method was used; thus, the request to answer the questionnaire was sent to social networks and the account owners forwarded the link. It was closed when no more responses were received from any respondent. The eligibility conditions were as follows: age between 18–35 years, having Romanian residency, and speaking Romanian as the mother tongue. The exclusion criteria are in opposition to those of the inclusion criteria: people who did not fit into the mentioned age range, were not native Romanian speakers, and who live abroad. The questionnaire was secured so that the same person could complete it only once. Respondents were informed that all answers were exclusively used for scientific purposes, in accordance with the requirements of the EU Regulations 2016/679 regarding the protection of personal data. The participants gave their informed consent before completing the questionnaires. Participants were informed about the purpose of the study. They were also informed that they could withdraw from the study at any time. We proceeded to the anonymous completion of the instruments as a method of controlling bias [13]. The completion time was 3 to 5 min. For the assessment of the test-retest reliability, 35 other participants completed the instruments in paper-and-pencil format at two- or three-weeks interval between the first completion and the retest. The latter were recruited from the dentist’s office.

### 3.4. Measures

1. Through its eight items, the DIS [1] measures one’s behavior regarding dental visits (the need for dental treatment in different circumstances) and one’s personal oral hygiene habits [1]. The total score is between 0 and 8. Sample items include the following: “If I had a very painful back tooth: a. I would prefer it to be taken out; b. I would prefer it to be left alone; c. I would prefer it to be filled” (item 2); and “I usually make an appointment to visit a dentist: a. When my dentist reminds me; b. At the end of my last appointment; c. When I think it is time to go for another check-up, d. Only when I think there is something wrong with my teeth” (item 4).

2. The questionnaire regarding one’s attitude towards oral health consisted of five questions:

- oral hygiene behavior (the daily frequency of tooth brushing with two choices: 1—two or more times a day; or 2—once a day or more rarely);
- eating behavior (the frequency of daily snacks: 1—one-two snacks between meals; or 2—three or more snacks);
- attendance (1—between six months and a year; or 2—more than a year);
- flossing (1—once a day; or 2—less than once a day);
- self-assessment of oral health (1—better than the average; 2—average, 3—worse than average).

### 3.5. The Socio-Demographic Data Collected

These types of data concerned the following aspects: (1) gender; (2) age; (3) education (primary, secondary, university, post-university); (4) residency (urban, rural); (5) work sector (public, private, other), and (6) geographical region (all eight regions of the country were included).

### 3.6. The Statistical Analysis of the Data

The statistical strategies were descriptive, and they were meant to capture the level of dental indifference (means, standard deviations, frequencies, and statistically significant differences). For the distributions of the scores, we calculated skewness and kurtosis in view of assessing normality. For normally distributed values the latter need to be between −1.00 and 1.00 [14]. Gender differences were calculated using the nonparametric Mann-Whitney U test. The reliability was assessed by means of internal consistency (α Cronbach and ω McDonald) and by means of test-retest every two-three weeks (intraclass correlation coefficient—ICC). In order to conclude that a unidimensional instrument has internal consistency and it is useful for research, we need a value of α and ω ≥ 0.70 [15]. For the assessment of ICC, we considered that <0.5 shows low reliability, the value between 0.50–0.5 shows moderate reliability, and >0.75 shows high reliability [16]. The validity of the scale consisted in associating the means scores of DIS with the answers to the five questions regarding oral health behavior. The purpose was to assess the capacity of DIS to discriminate between healthy and unhealthy oral habits. The SPSSv22 (IBM, New York, NY, USA) and JASP 0.16.10 (University of Amsterdam, Amsterdam, the Netherlands) programs were used.

## 4. Results

### 4.1. Socio-Demographic Characteristics

The analyzed group was made of 660 participants with a mean age of 30.47 years (Std.Dev. = 14.37); 30.30% (200) of them were male subjects (Mean_age_ = 29.52; Std.Dev. = 15.50) and 69.69% (460) were female (Mean_age_ = 31.07; Std.Dev. = 15.35). As for education, 49.69% (328) of participants were university graduates, 27.57% (182) had a secondary education, and 22.72% (150) of respondents were post-university graduates. Most participants (80.90%) (534) came from an urban area and 19.09% (126) lived in a rural area. As for the working environments, most of the participants (35.75%) (236) worked in the public sector, 33.63% (222) in the private sector, 13.93% (92) were freelance, and 16.66% (110) were unemployed. Most of the respondents (30.8%) were from the area of the country’s capital, 14.9% come from the southeast, 13.4% came from South Muntenia, 13.93% were from the northeast, 10.4% lived in the southwest, 7.27% resided in the central region, and 9.4% were from the west and northwest of the country, respectively.

### 4.2. Descriptive Analysis

For the eight questions of the Romanian version of the DIS, the values of the skewness indicator (Table 1) were between 0.03 and 3.56, while the absolute values of the kurtosis indicator were between −1.91 and 11.11. The values of the respective indicators show the non-normality of data distribution.

The average of the total scores to DIS is 2.45 (Std.Dev. = 1.32). The comparison with the average score obtained in the case of 600 Scottish residents (Mean _DIS total score_ = 3.1; Std.Dev. = 1.9) [8] and Norwegian residents (Mean _DIS total score_ = 3.0) [10], suggests that the dental indifference in the case of the present sample is lower in comparison to that of the other populations (with the caveat that one should cautiously compare individuals from different cultural environments).

**Figure 1:**
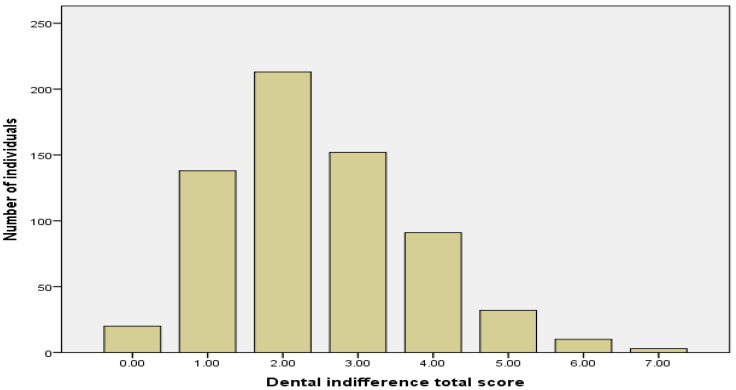
The frequency of DI at the level of the entire group.

Figure 1 highlights that most individuals were found to have average levels of DI. The effect of gender and education on DI scores was then calculated. There were no significant differences for gender for the total score of DIS: Mean Rank_males_ = 345.72; Mean Rank_females_ = 324.62; U = 43,157.00; z = 1.19; *p* = 0.179. Conversely, the DI level makes the difference between the subjects with secondary studies (Mean Rank = 280.64) and those with university studies (Mean Rank = 247.73) (U = 26,259.00; *p* = 0.015) (Table 2).

### 4.3. Reliability

The calculation of consistency coefficients for the eight questions show values of 0.37 (95%CI = 0.31–0.44) in the case α Cronbach and 0.39 (95%CI = 0.32–0.46) for ω McDonald (Table 3). For 35 patients with the mean age 43.40 (Std.Dev. = 16.34), we conducted the retest after two or three weeks. The coefficient ICC (3.1) between test and retest in the case of the eight questions is 0.60 (95%CI = 0.38–0.75) (*p* < 0.001) for the total score of DIS. The Kappa values were calculated for the eight questions. As Table 3 shows, five questions have Kappa values over 0.40 (between 0.44 and 0.79). Question 6 has the lowest Kappa value (kappa = −0.06).

### 4.4. Dental Indifference and Oral Health Behavior

Prior studies found that the global DIS score is associated with inadequate personal oral hygiene behavior (a low frequency of brushing and flossing), the frequency of dental visits (missed appointments) [10], as well as pain or discomfort of the mouth [8]. That is why we considered that the average score for DI could make a difference between the healthy and the unhealthy oral health behaviors. Table 4 shows those that are significant.

## 5. Discussion

The study aimed to analyze the properties of DIS for the Romanian population. The present research has shown that the global score of DI makes a difference with regard to one’s behavior towards oral health. Thus, individuals with higher DI brush less than twice a day, do not control their eating habits (they have more than three snacks between meals), rarely visit the dentist, and use dental floss less than once a day. There are similarities in our findings to a study on subjects from New Zeeland which showed that people with high DI scores visit the dentist when they had dental problems and not for regular check-ups, they brush less than once a day, they use floss very rarely and they feel embarrassed about the state of their teeth [8]. The result corroborates the result of a study which found that the DI level for treatment is associated with oral hygiene behaviors such as flossing and brushing before bed [11]. Unlike prior studies that found higher DI for young people [1,8] and male subjects [1], in the present study there were no DI score differences that were dependent on gender. However, the DI scores indicated a distinction between the individuals with a secondary level of education and those with a university education.

The low reliability of internal consistency coefficients (α = 0.37; ω = 0.39) and the moderate reliability of ICC coefficients for test-retest (ICC = 0.60) shows that the scale needs to be used with caution for the Romanian adult population. In two prior studies, the value of α coefficient for DIS was >0.70 [1,11]. The present results get us closer to the study of Skaret et al. (2000), who found low reliability both in terms of the level of internal consistency (α = 0.35) and retesting (r = 0.43 for the eight questions) [10]. We agree with the explanations according to which the questions of the scale do not form a unitary concept for what DI could signify [10]. There are similarities and dissimilarities with regard to the Kappa values. In the study carried out on Norwegians [10], the Kappa values were between 0.10 and 0.70, and in the present study they are between −0.06 and 0.79. Question 8 obtained the best score in both studies (0.79—the present study; 0.70—the study of Skaret et al., 2002) [10]. Question 6 (dental appointments) registered a slightly negative Kappa value, which suggests a very low agreement between the respondents with regard to the fact that they have very different values or interpretations regarding appointments at the dental office: it seems that most of the respondents attended their appointments, while others skipped appointments for various reasons related to negligence.

Another aspect that makes us reluctant to use the scale is the quantification of scores by the specialist. Although the scale is easy to apply and it is not affected by the observation process [8,17], scoring instructions that differ for each question make the scoring process time-consuming and makes it difficult to use it in practice. It is worthy of note that DIS did not benefit from a qualitative study in its preliminary stages of development, which could allow for the study of the DI phenomenon [8].

The results obtained lead to the conclusion that DIS cannot be used as a single instrument in the assessment of the behaviors and the attitudes towards oral health. Instead, a corpus of instruments is required. For example, we consider it useful to use the DIS alongside scales such as the Dental Neglect Scale that measure valid constructs which reference the failure in providing oral care (hygiene, adequate diet, professional care) and the inability to seek treatments for dental issues [18]. Because it is useful to understand the factors that determine attitudes and behaviors of dental indifference, it is equally important to associate DIS with instruments such as the Oral Health Values Scale, which assesses how much individuals invest in their dental health; for example, how much they value professional dental care and the appearance and retention of their natural teeth [19].

### Limits of the Study

One of the limits of the present study is the asymmetry of the sample, as it included more female than male subjects. Another limitation was the non-probabilistic sampling technique used for collecting data, and therefore the results may not be generalizable. The snowball sampling makes data collection easier, but one must not forget that this technique entails the possibility that respondents recommend people with characteristics similar to their own [20].

## 6. Conclusions

The obtained findings on the studied sample lead us to consider that DIS is a scale that should be used with caution due to its relatively low fidelity with regard to the test-retest and internal consistency indices. These characteristics of the DIS make it questionable regarding its use in research and practice. Further research is needed in order to examine the validity of DIS in the assessment of interventions regarding the promotion of oral health. Supplementary research, such as qualitative requirements and experimental manipulation, are important for a deeper understanding of the DI construct. It is likely that the improvement of DIS by reconsidering it as a self-reporting scale would increase the degree of accuracy in measuring the behaviors and attitudes related to dental indifference. At the same time, it is important to analyze the psychometric properties of the scale for other age categories and to compare them with the data of the present study. A dental indifference measure is absolutely useful for identifying patients with increased levels of indifference. Once this is achieved, it is useful to raise awareness of its implications in relation to oral health status. This means that educational programs and preventive strategies must be implemented quickly. In addition to this aspect, it is important to have tools that explore the deeper causes that determine dental neglect, from fears of different types of treatment to the belief in good personal hygiene so that there is no need for frequent visits to the dentist.

## Figures and Tables

**Table 1:** The descriptive analysis of the items (means, standard deviation, skewness and kurtosis).

Items	M	Std.Dev	Min.–Max.	Skewness	Kurtosis
1	0.87	0.32	0–1	−2.32	3.43
2	0.51	0.50	0–2	−0.03	−1.91
3	0.16	0.37	0–2	2.00	2.56
4	0.30	0.45	0–1	0.87	−1.24
5	0.36	0.48	0–1	0.56	−1.68
6	0.06	0.24	0–1	3.56	10.74
7	0.10	0.30	0–1	2.63	4.95
8	0.06	0.24	0–1	3.61	11.11
DIS total score	2.45	1.32	0–7	0.56	0.22

M—Mean; Std.Dev.—Standard deviation; Min.–Max.—minimum and maximum values.

**Table 2:** Gender and education differences (means and Mann-Whitney coefficient).

Mean Rank	Gender		Education	
	Males	Females	Secondary Level	University Level
DIS total score	345.72	324.62	280.64	247.73
Mann-Whitney U	43,157.00		26,259.00	

**Table 3:** ICC for the eight questions of DIS.

Test-Retest	N	Kappa Values	% Agreement	
			0	1
DIS1	35	0.68	34	62
DIS2	35	0.47	62	25
DIS3	35	0.37	82	8
DIS4	35	0.60	42	48
DIS5	35	0.44	65	22
DIS6	35	−0.06	94	5
DIS7	35	0.34	80	17
DIS8	35	0.79	82	17
DIS total score: α = 0.37 ω = 0.39				

**Table 4:** The mean scores for dental indifference depending on one’s behavior towards oral health.

Oral Health Behavior	Mean DIS (Std.Dev.)	*p*<
Daily frequency of tooth brushing		
Two or more times a day	2.71 (1.30)	0.001
Once a day or more rarely	3.43 (1.46)	
Eating behavior		
Large number of snacks between meals	2.71 (1.30)	0.001
Small number of snacks between meals	2.10 (1.22)	
Dental attendance		
6 months–1 year	2.22 (1.18)	0.001
More than a year	3.37 (1.18)	
Flossing		
Once a day	1.92 (1.10)	0.001
Less than once a day	2.71 (1.31)	
Self-assessment of oral health		
Better than the average	2.27 (1.22) *	0.001
Average	3.31 (1.33)	
Worse than the average	4.00 (0.80) **	0.005

* significant difference between group 1 and group 2; ** a significant difference between group 1 and 3; differences of the average values of DI depending on diet, on the frequency of brushing, the frequency of flossing, and the frequency of dental visits.

## Data Availability

The data presented in this study are available from the corresponding authors upon reasonable request.

## Appendix A

**Table A1:** The English and Romanian versions of the DIS administered in the current study.

Items from the Original English Version	Items from the Final Romanian Version
1. I usually use: A toothbrush to clean my teeth Floss or a special brush to clean my teeth Disclosing tablets to check my teeth are clean	1. Folosesc de obicei: O periuță dentară pentru a-mi curăța dinții Ață dentară sau o perie specială pentru a-mi curăța dinții Tablete pentru a verifica dacă dinții mei sunt curați
2. At present: I think there is something wrong with my teeth but it is not bad enough to go to a dentist I think there is something wrong with my teeth and I intend to see a dentist about it soon I am going for a check up in the next year I do not think I need any treatment so I am not planning on going to a dentist just now	2. În momentul de față: Mă gândesc că dinții mei au probleme, care nu sunt, însă, atât de mari încât să merg la medicul stomatolog Mă gândesc că am probleme cu dinții și intenționez să merg la medicul stomatolog cât de curând posibil Voi merge la control anul viitor Nu cred că este necesar tratamentul și nu intenționez să merg la medicul stomatolog chiar acum
3. If I lost a filling in a back tooth and it did not hurt: I would immediately arrange to go to a dentist I would wait to see if it started hurting or got any worse before going to a dentist It would not be a problem I would not see a dentist about it	3. Dacă aș pierde o plombă de pe dintele din spate și nu mă doare: Mi-aș aranja imediat să merg la medicul stomatolog Aș aștepta să văd dacă începe să mă doară sau se întâmplă ceva și mai rău înainte de a merge la medicul stomatolog Nu ar fi o problemă și nu aș merge la medicul stomatolog pentru atâta lucru
4. I usually make an appointment to visit a dentist: When my dentist reminds me At the end of my last appointment When I think it is time to go for another check-up Only when I think there is something wrong with my teeth	4. Îmi fac programare pentru a merge la medicul stomatolog: Atunci când acesta îmi aduce aminte La finalul ultimei programări Atunci când consider că este momentul pentru un nou control Atunci când consider că ceva nu este în regulă cu dinții mei
5. If my gums bled, but they didn’t hurt: It would not be a problem. I would not see a dentist about it I would immediately arrange to go to a dentist I would wait to see if it started hurting or got any worse before going to a dentist	5. Dacă gingiile mi-ar sângera, dar nu m-ar durea: Asta nu ar fi o problemă și nu aș merge la medicul stomatolog pentru atâta lucru Aș merge imediat la medicul stomatolog Aș aștepta să văd dacă încep să mă și doară sau să văd dacă se întâmplă ceva și mai rău înainte de a merge la medicul stomatolog
6. About all your dental appointments during the last 5 years: I have not made a dental appointment in the last 5 years During the last 5 years I have forgotten to go to a dental appointment During the last 5 years I have only missed an appointment through illness or another unavoidable reason During the last 5 years I have never missed a dental appointment During the last 5 years I have cancelled a dental appointment because the problem went away	6. Referitor la programările dvs. la cabinetul dentar din ultimii 5 ani, puteți spune că: Nu mi-am făcut nici o programare în ultimii 5 ani Am uitat să mă prezint la una din programări în ultimii 5 ani Nu m-am prezentat la una din programări din motive de boală sau alte situații pe care nu am putut să le evit M-am prezentat la toate programările în ultimii 5 ani In ultimii 5 ani am anulat o programare deoarece problema a dispărut de la sine
7. If I had a very painful back tooth: I would prefer it to be taken out I would prefer it to be left alone I would prefer it to be filled	7. Dacă aș avea un dinte din spate foarte dureros: Aș prefera să fie scos Aș prefera să nu fie tratat Aș prefera să fie plombat
8. I would say that my main reason for not going to a dentist for a check-up would be: Because I think treatment is painful Because it takes too long to go to a dentist Because I feel anxious or worried about going Because I cannot see the point of visiting for a check-up given Because my dentist makes me feel guilty about the state of my teeth Because it costs too much Because I have no time to go to a dentist I do not put off going. I attend for regular check-ups	8. Aș spune că principalul motiv pentru care nu merg la medicul stomatolog pentru control ar fi: Deoarece cred că tratamentul este dureros Deoarece programarea durează prea mult Deoarece sunt anxios sau îngrijorat în legătură cu acest lucru Deoarece nu înțeleg rostul vizitei pentru un control de rutină Pentru că medicul stomatolog mă face să mă simt vinovat de starea dinților mei Deoarece costă prea mult Pentru că nu am timp să merg la cabinetul stomatologic Nu amân, mă prezint la controale regulate
